# miR-205 Enhances Sensitivity to Genotoxic Agents in HNSCC Cells and Blocks Sphingosine Kinase 2 Action in Tumorigenicity

**DOI:** 10.1021/acsomega.5c06726

**Published:** 2025-12-11

**Authors:** Thaís Moré Milan, Gabriel da Silva, Graziella Ribeiro de Sousa, Andréia Machado Leopoldino

**Affiliations:** Department of Clinical Analyses, Toxicology and Food Sciences, School of Pharmaceutical Sciences of Ribeirão Preto, 67782 University of São Paulo, Av. Do Café, S/N, Ribeirão Preto, São Paulo 14040-903, Brazil

## Abstract

Head and neck squamous cell carcinoma (HNSCC) exhibits alterations in sphingolipids, sphingosine kinase 2 (SK2), and microRNAs (miRNAs), including miR-205. Resistance to therapy, such as cisplatin and radiation, often leads to recurrence and metastasis. This study investigates the role of SK2 in resistance to DNA damage induced by cisplatin and UV light, as well as the potential of miR-205 to reverse this resistance in HNSCC cells. For this purpose, we adopted SK2 overexpression and SK2 knockdown associated with miR-205 mimic in oral carcinoma keratinocyte cell lines and an in-house established squamous carcinoma primary cell line (LMSCC-03) as models. Resistance to UV and cisplatin was evaluated by using orosphere and clonogenic assays. Western blot analysis was performed to investigate how miR-205 mediates SK2 effects on EMT proteins following exposure to genotoxic agents. NOK-SK2 cells were more resistant to UV exposure than NOK-Ø cells in clonogenic and orosphere assays. Conversely, SK2 knockdown in HN13 cells increased sensitivity to UV exposure, indicating that SK2 confers resistance to UV damage. Interestingly, miR-205 overexpression, in combination with UV or cisplatin exposure, inhibited SK2-mediated resistance to these genotoxic agents in HN12-SK2 and NOK-SK2 cells and decreased the levels of proteins involved in the epithelial-mesenchymal transition (EMT). In LMSCC-03, overexpression of miR-205 significantly decreased cellular viability and clonogenic potential. Moreover, combining cisplatin treatment with miR-205 overexpression abolished the clonogenic potential in LMSCC-03 cells. Our study reveals, for the first time, that miR-205 can be an alternative for sensitization to treatment with genotoxic agents, opening a new and promising strategy for highly malignant HNSCC and overcoming cisplatin resistance.

## Graphical abstract

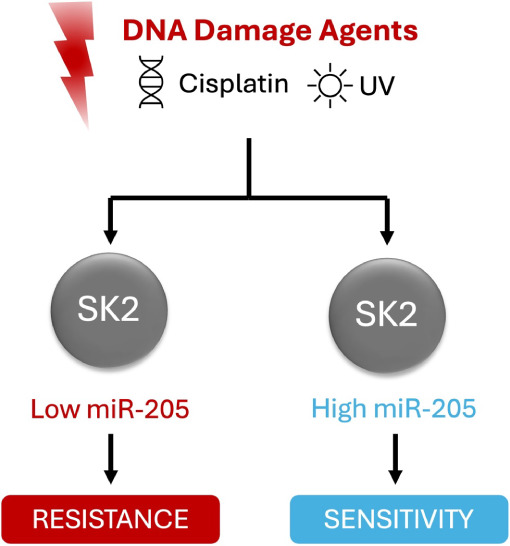

## Introduction

Head and neck squamous cell carcinoma (HNSCC) is the seventh most common cancer in the world, associated with high mortality, lower survival rates, and over 60% of recurrences and metastases.^1–3^ By 2040, its incidence is estimated to increase by 40%.^4^ Alcohol and tobacco use, human papillomavirus (HPV) infection, diet, and hereditary predisposition are some of the risk factors associated with HNSCC.^5^ The standard treatments for HNSCC include surgical resection, followed by adjuvant radiation or chemotherapy/radiation, depending on the disease stage.^6^ Cisplatin, a platinum-based drug, is used as the first-line treatment in conjunction with radiotherapy (RT). Lamentably, cancer cells often develop resistance to systemic chemotherapy (CT) and RT, leading to treatment failure or even cancer recurrence.^7^ Therefore, investigating the molecular mechanisms of chemoradiotherapy resistance may lead to improved outcomes for HNSCC.

In many types of cancer, the abnormal expression of multiple miRNAs has been linked to cellular proliferation, apoptosis, metastasis, migration, and drug resistance.^8^ In HNSCC, deregulated miRNAs are observed in the tumoral cells and saliva of patients, making them potential biomarkers for the progression and development of HNSCC.^9,10^ Moreover, growing evidence has shown that miRNAs play critical roles in regulating the DNA damage response (DDR) and repair pathways, thereby orchestrating mechanisms of chemoradioresistance.^11^

The miRNA-205 on chromosome 1q32.2 is up- or downregulated in various cancers.^12^ Previous findings have revealed that high levels of miR-205 enhance the sensitivity of breast cancer cells to TAC (docetaxel, doxorubicin, and cyclophosphamide) chemotherapy by suppressing both VEGFA and FGF2, leading to increased cell apoptosis.^13^ Another study reported miR-205 as a suppressor of tumorigenesis, with its overexpression enhancing apoptosis and resensitizing human liver cancer cells to doxorubicin.^14^ Interestingly, our previous studies identified miR-205 as being downregulated in oral keratinocyte and carcinoma cell lines with high levels of sphingosine kinase 2.^15^

Sphingosine kinase 2 has been linked to a broad spectrum of biological functions, including cell survival, cell cycle progression, apoptosis, and senescence.^16^ Our prior studies have demonstrated SK2 as an essential inducer of transformation in oral cancer cells and the xenograft tumor model.^17^ In addition, SK2 contributes to drug resistance in oral squamous cell carcinoma (OSCC) cells by enhancing DNA damage repair pathways.^18^

Our present work demonstrates that the overexpression of miR-205 in NOK-SK2 and HN12-SK2 cells restores sensitivity to UV light and cisplatin. This is accompanied by a reduction in stemness properties, clonogenic potential, and EMT markers, such as ZEB2 and STAT3, which are reported as targets of miR-205. In conclusion, increasing miR-205 can overcome DNA damage resistance, at least when sustained by increased SK2 levels, highlighting a potential new therapeutic strategy to improve HNSCC outcomes.

## Results

### SK2 Promotes UV Resistance and Maintains Cancer Cell Stemness in Oral Keratinocytes and HNSCC Cells

Previous studies from our group and others have shown that SK2 overexpression modulates the DNA damage response^18^ and enhances the stemness program.^17^ To explore how SK2 activation increases cancer stemness in HNSCC cells and mediates resistance to DNA damage, we used orosphere cultures derived from nontumor keratinocytes NOK-Ø/NOK-SK2 and HN13-Ø/HN13-shSK2 cell lines, with or without UV irradiation (Figure S1A,B). In NOK-Ø cells, the number of cell spheres was entirely abrogated in NOK-Ø (13.0-fold, *p* < 0.001) when exposed to UV (16.0-fold; *p* < 0.001), in contrast to the NOK-SK2 spheres (1.74-fold; *p* < 0.001), which demonstrated that SK2 can confer resistance to DNA damage (Figure 1A). To assess cellular plasticity and metastatic potential, we transferred the spheres from the previous experiment onto adhesion plates and found that NOK-SK2 attached to plates and grew as adherent monolayer cells following UV exposure (Figure 1B). The results of colony formation showed a significant reduction in the number of colonies in NOK-Ø (40.0-fold, *p* < 0.05) compared to NOK-SK2 (6.38-fold, *p* < 0.001), indicating greater sensitivity to UV exposure (Figure 1C). Interestingly, single-sphere analysis displayed a markedly elevated length of NOK-SK2 (1.6-fold; *p* < 0.0001) versus NOK-Ø (2.6-fold; *p* < 0.0001) (Figure 1D) exposed to UV. Conversely, SK2 knockdown in HN13 cells not only significantly inhibited the rate of sphere formation compared to the mock control but also inhibited the attachment of sphere cells (HN13-Ø: 10.0-fold versus HN13-shSK2: 60.4-fold) (Figure 1E,F). Similarly, colony formation assays demonstrated that SK2 downregulation substantially reduced the colony-forming ability (18.1-fold, *p* < 0.01) and decreased sphere diameter (2.75-fold, *p* < 0.001) (Figure 1G,H). These results suggest that SK2 is crucial in sustaining proliferation and resistance to DNA damage in reprogrammed orospheres following UV exposure.

**Figure 1:**
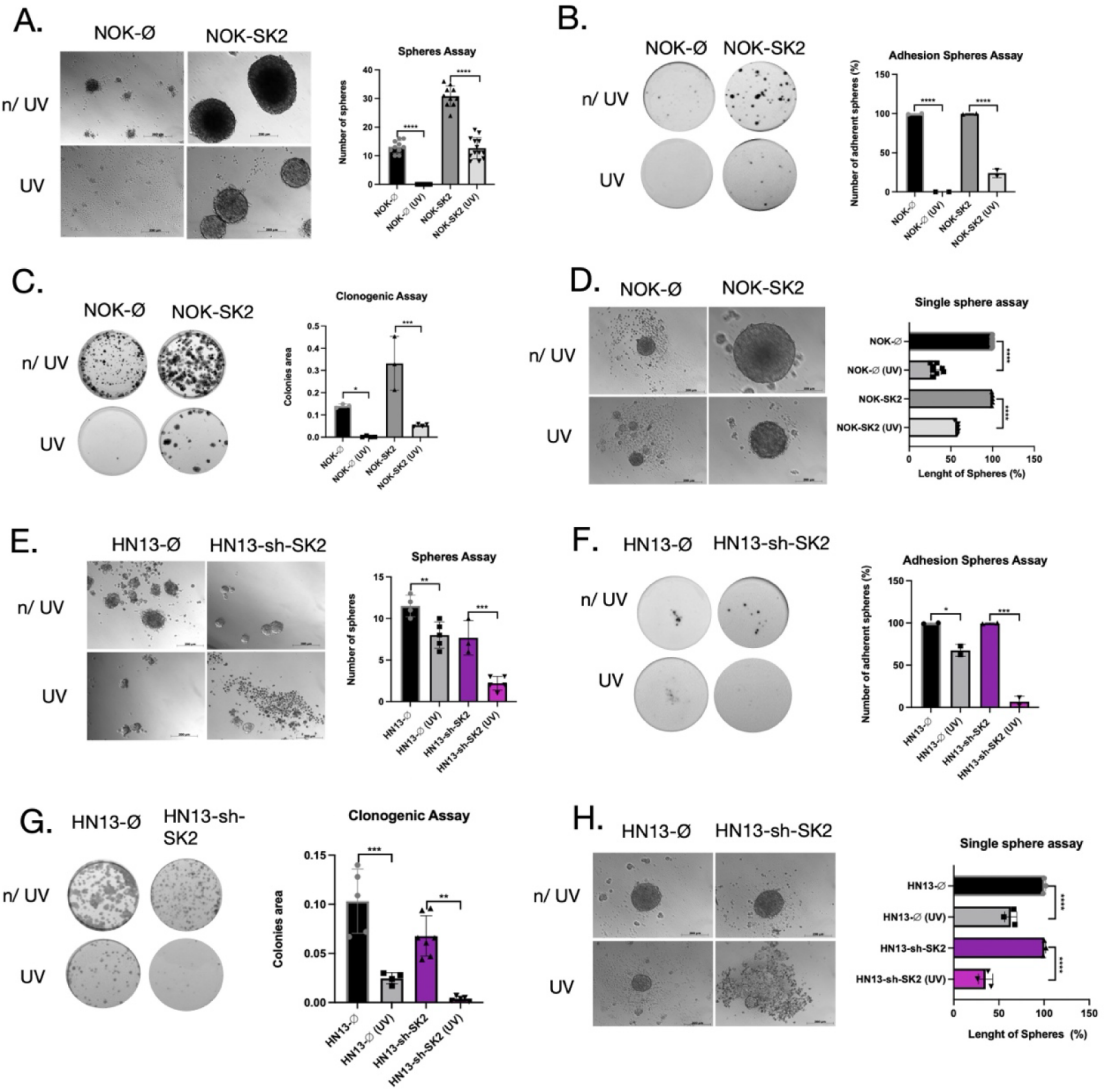
Effect of UV exposure on SK2-overexpressing and knockdown cells. (A) Orosphere formation assay on NOK-Ø and NOK-SK2 cells to evaluate the self-renewal ability of the cells after UV radiation. (B) Adhesion sphere assay on NOK-Ø and NOK-SK2 cells post-UV exposure. (C) Colony formation assay to determine the effect of UV. (D) Length of spheres generated by NOK-Ø and NOK-SK2 cells from exposure to UV. (E) Sphere-forming assay used to determine the number of spheres in the HNSCC SK2-knockdown cell line: HN13-shØ and HN13-shSK2 cells. (F) Adhesion sphere assay on HN13-shØ and HN13-shSK2 cells. (G) Sphere length comparison of HN13-shØ and HN13-shSK2 cells after UV exposure. Images of spheres were obtained with bright light. Scale bar: 200 μM. *p < 0.05, **p < 0.01, ***p < 0.0001.

### Effect of miR-205 Overexpression on DNA Damage Agents in Response to Oral Keratinocytes and Carcinoma Cell Lines

In a recent study, we observed that miR-205 is significantly downregulated in SK2-overexpressing cells. However, when miR-205 was overexpressed, it resulted in reduced cell migration, sphere formation, and prevention of epithelial-mesenchymal transition (EMT).^15^ Therefore, we decided to investigate whether the overexpression of miR-205 in NOK-SK2 and HN12-SK2 cell lines could sensitize SK2-mediated resistance to DNA damage caused by UV exposure. As shown in Figure 2A, a 1.43-fold decrease in sphere formation was observed in the NOK-SK2 pcDNA cells (control) after UV radiation compared to unexposed cells (*p* < 0.0001). On the other hand, a 2.75-fold decrease in the number of spheres was observed in NOK-SK2 cells overexpressing miR-205 (NOK-SK2 pcDNA/miR-205) after UV exposure compared to the control (*p* < 0.0001). Similar results were observed when transferring the spheres from the previous experiment onto adhesion plates after exposure to UV, showing a significant and more pronounced decrease in adhered spheres with miR-205 overexpression and exposure to UV radiation (*p* < 0.001) (Figure 2C).

**Figure 2:**
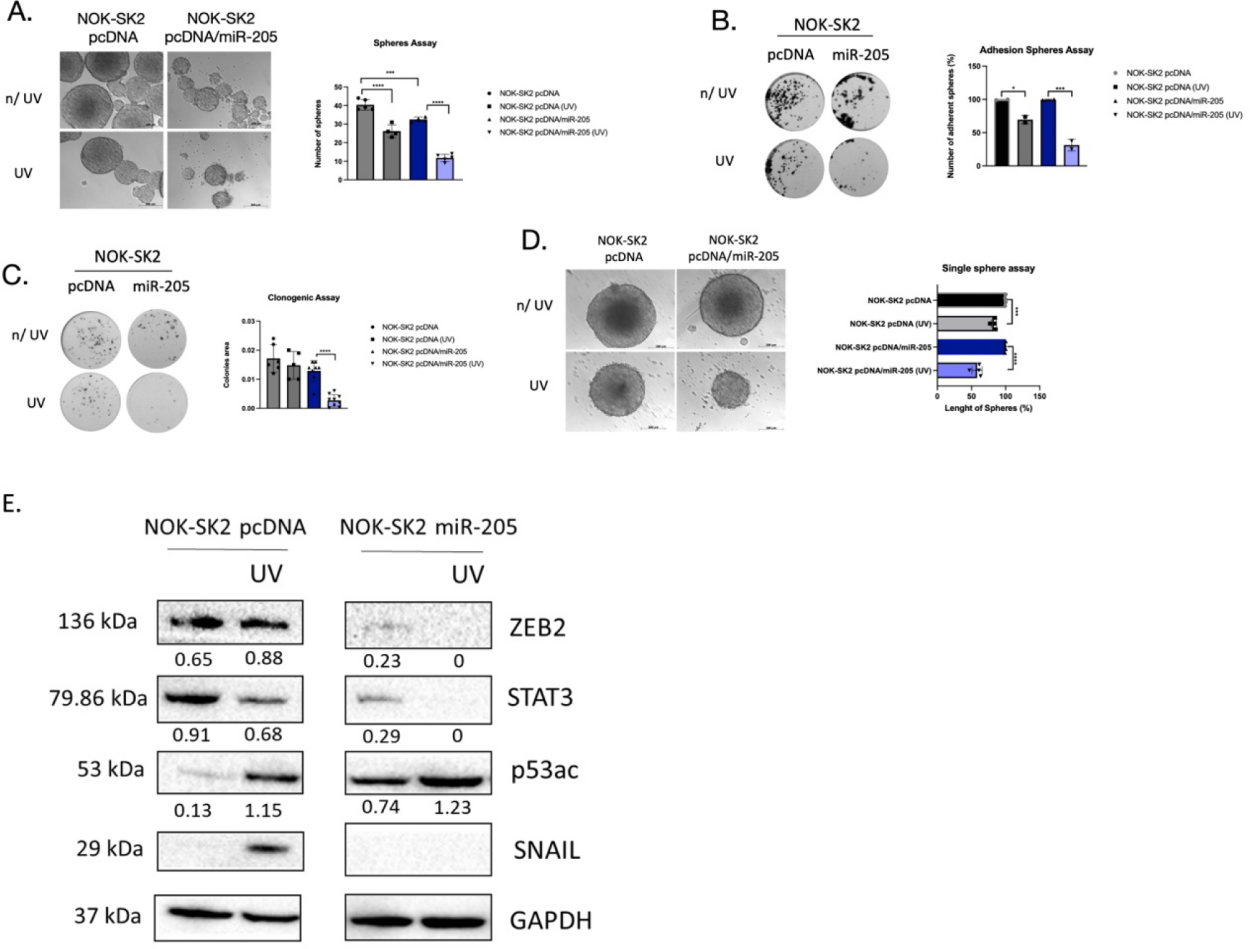
miRNA-205 promotes radiosensitivity in SK2-expressing cell lines. (A) Orospheres formed on miR-205 overexpression in NOK-SK2 (pcDNA-miR-205) cells compared to control NOK-SK2 (pcDNA) post-UV exposure. Adhesion sphere assay (B); colony formation was determined by colony formation assay (C); and single spheres (D) on NOK-SK2 (pcDNA-miR-205) cells compared to control NOK-SK2 (pcDNA). (E) Western blot analysis for the protein levels of ZEB2, STAT3, p53Ac, and Snail. Images of spheres were obtained with a microscope. *p < 0.05, **p < 0.01, ***p < 0.0001.

As shown in Figure 2D, corroborating our results, the single-sphere assay revealed a 1.20-fold decrease in sphere length in NOK-SK2 pcDNA after exposure to UV (*p* < 0.001). However, in NOK-SK2 cells overexpressing miR-205, greater sensitivity to UV radiation was observed, with a significant 1.72-fold decrease in sphere length (*p* < 0.0001). In Figure 2B, similar results were observed using the clonogenic assay. A 1.12-fold decrease in clonogenic potential was seen for the NOK-SK2 pcDNA after UV exposure, while a significant 4.78-fold decline was observed in the miR-205 overexpression (*p* < 0.0001).

Evaluating proteins involved in epithelial-mesenchymal transition (EMT) and cancer stem cell properties, we observed a significant decrease in ZEB2 with miR-205 overexpression (NOK-SK2 pcDNA/miR-205) compared to the control (NOK-SK2 pcDNA). Interestingly, after exposure to UV radiation, ZEB2 protein expression was abolished with the overexpression of miR-205.

Assessing STAT3, similar results were observed, with a significant decrease in the NOK-SK2 pcDNA/miR-205 line, showing no protein accumulation after UV exposure. At the same time, the NOK-SK2 pcDNA control line exhibited a slight decrease after UV exposure (Figure 2E). We also observed an increase in p53ac Lys382 in the NOK-SK2 pcDNA/miR-205 line compared to the control, which was more activated after UV exposure. However, the control cell line showed decreased levels of p53ac, with an increase only after UV exposure (Figure 2E).

We also tested whether miR-205 could reverse SK2-mediated resistance in HN12-SK2 cells and observed a 4.52-fold reduction (*p* < 0.0001) in sphere formation in the control (HN12-SK2 pcDNA) after UV exposure. Notably, miR-205 overexpression (HN12-SK2 pcDNA/miR-205) resulted in an 8.91-fold reduction (*p* < 0.0001), indicating that miR-205 sensitizes SK2-mediated UV resistance in oral carcinoma cells (Figure 3A). Similar changes were seen in the single sphere assay, with a 2.20-fold (*p* < 0.0001) reduction in sphere length in the control line and a 7.70-fold reduction (*p* < 0.0001) in the miR-205 overexpression line after UV exposure (Figure 3B), suggesting increased sensitivity to DNA damage.

**Figure 3:**
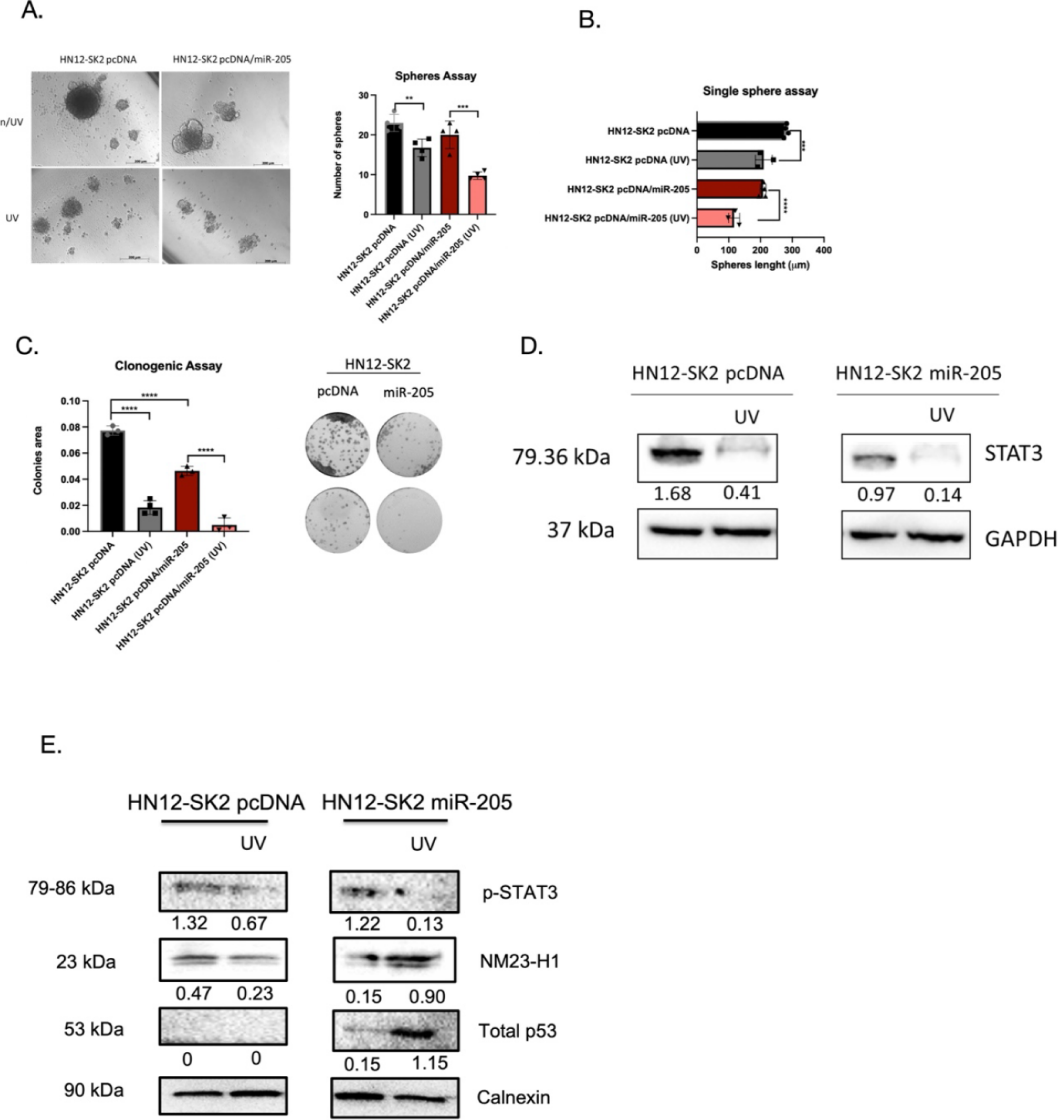
MiRNA-205 reverses the resistant phenotype in HN12-SK2 cells. (A) miR-205 overexpression in HN12-SK2 (pcDNA-miR-205) cells compared to control HN12-SK2 (pcDNA) was more sensitive to post-UV exposure. (B) Sphere-forming assay used to determine the length after UV radiation of HN12-SK2 (pcDNA-miR-205) versus HN12-SK2 (pcDNA). (C) Colony formation assay shows abrogated colonies in HN12-SK2 (pcDNA-miR-205) treated with UV radiation. (D) Western blot analysis for the protein levels of STAT3. E) Western blot analysis for the protein levels of p-STAT3, NM23-H1, and total p53.

Through the clonogenic assay, exposure to UV light was again sufficient to reduce the colony area in the HN12-SK2 pcDNA cells (3.98-fold)­(*p* < 0.0001). However, the most significant reduction occurred with the overexpression of miR-205 (HN12-SK2 pcDNA/miR-205) (9.2-fold) (*p* < 0.0001) (Figure 3C). The STAT3 protein levels were markedly decreased in the miR-205 overexpression (HN12-SK2 pcDNA/miR-205) after exposure to UV radiation. In the control cell line (HN12-SK2 pcDNA), the STAT3 protein expression was also decreased after exposure to UV radiation; however, the most significant reduction occurred in the HN12-SK2 pcDNA/miR-205 cell line (Figure 3D). Similar to STAT3, p-STAT3 levels were decreased after UV radiation, mainly in combination with miR-205 (Figure 3E). To further investigate DNA damage, we analyzed NM23-H1, a metastasis suppressor gene linked to DNA repair and UV-induced damage.^19^ An increased protein level was observed with the combination of miR-205 and UV radiation (HN12-SK2 pcDNA/miR-205 + UV). Additionally, we observed that the combined treatment of miR-205 overexpression and UV exposure in HN12-SK2 cells increased p53 protein levels (Figure 3E). Since MDM2 is a validated target of miR-205,^20^ this p53 recovery or reactivation may be linked to the downregulation of MDM2, accompanied by the reduction of p53 ubiquitination and degradation.

Images of spheres were obtained using a microscope. *p < 0.05, **p < 0.01, ***p < 0.0001.

### miR-205 Enhances the Sensitivity of SK2 Induced by Cisplatin in the HNSCC Cell Lines and a Primary OSCC Lineage Established In-House

Radiation therapy or chemotherapy is typically administered when a patient is diagnosed with HNSCC at an advanced stage. However, chemoresistance remains a significant challenge to improving survival rates.^6^ Given our observation that miR-205 reprograms the DNA damage response in SK2-overexpressing HNSCC cell lines, we speculated whether miR-205 could reverse therapeutic resistance to cisplatin. As shown in Figure 4A,B, treating orospheres of miR-205-transfected NOK-SK2 and HN12-SK2 cells with different concentrations of cisplatin for 5 days resulted in a significant decrease in cell viability (*p* < 0.001). Next, to determine whether miR-205 affects colony formation following cisplatin treatment, we used NOK-SK2 pcDNA/miR-205 and HN12-SK2 pcDNA/miR-205 cell lines. In this experiment, miR-205 transfection in NOK-SK2 and HN12-SK2 cells significantly reduced colony formation in a dose-dependent manner (1–3 μM) compared to pcDNA-transfected controls, which were less sensitive to cisplatin treatment (Figure 4C,D). These results suggest that miR-205 may reverse chemoresistance to cisplatin mediated by SK2.

**Figure 4:**
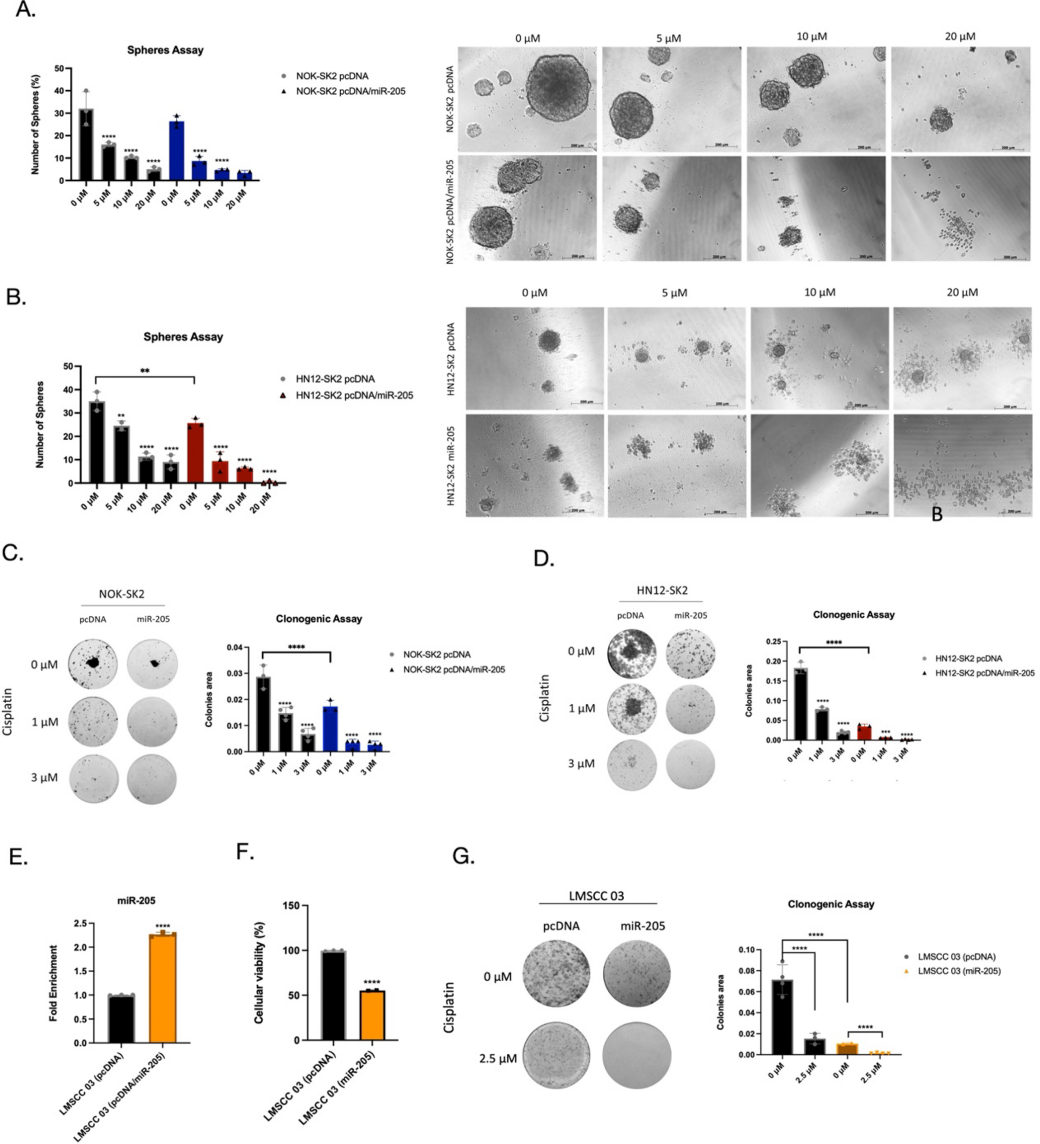
Effect of miR-205 overexpression on cisplatin and UV radiation sensitivity in HNSCC cell lines and primary cells. (A–B) Treatment of miR-205-transfected NOK-SK2 and HN12-SK2 orospheres with different concentrations of cisplatin led to a significant decrease in cell viability (*p* < 0.001). (C–D) Clonogenic assays demonstrated that miR-205 transfection in NOK-SK2 and HN12-SK2 cells significantly reduced colony formation in a dose-dependent manner (1–3 μM cisplatin) compared to pcDNA-transfected controls, indicating enhanced sensitivity to cisplatin treatment. (E) The establishment of miR-205 overexpression in LMSCC-03 cells was confirmed by qPCR (*p* < 0.0001). (F) Cellular viability assays showed a significant decrease in LMSCC-03 cell viability following miR-205 overexpression (*p* < 0.0001). (G) Clonogenic potential was markedly reduced in LMSCC-03 cells after miR-205 overexpression, treated with 2.5 μM cisplatin, compared to pcDNA control cells (*p* < 0.0001).

To further elucidate the effect of miR-205 and cisplatin, we evaluated a primary cell line established in-house from a tongue squamous cell carcinoma (LMSCC-03). First, we subjected LMSCC-03 to miR-205 overexpression, which was confirmed by qPCR (*p* < 0.0001) (Figure 4E) and led to a significant decrease in cellular viability (*p* < 0.0001) (Figure 4F). The clonogenic potential of LMSCC-03 cells was significantly diminished following the overexpression of miR-205, resulting in a remarkable 7.1-fold reduction (*p* < 0.0001). Furthermore, while treatment with 2.5 μM cisplatin significantly decreased clonogenicity in LMSCC-03 control cells by 4.73-fold, the combination of miR-205 overexpression and cisplatin treatment resulted in a more pronounced reduction (10-fold, *p* < 0.0001) (Figure 4G).

## Discussion

A recent study by our research group demonstrated, for the first time, that the overexpression of sphingosine kinase 2 (SK2) is sufficient to transform nontumoral oral keratinocyte cells into tumorigenic cells.^17^ Moreover, SK2 has been implicated in the progression of various cancers, such as acute lymphoblastic leukemia (ALL) and non-small cell lung cancer (NSCLC).^21^ Notably, another study from our research group found that NOK-SK2 cells exhibit increased activation of pathways associated with DNA damage resistance.^18^ Here, we first confirmed the stemness profiles and clonogenic potential of NOK-SK2 compared to those of NOK control cells, as well as HNSCC cells (HN13-Ø and HN13-sh-SK2), after UV exposure. It is well known that UV exposure causes DNA damage.^22^ As expected, cells with SK2 accumulation exhibit higher resistance to UV, as shown by decreased sphere/sphere length and colony formation in oral keratinocyte cells without SK2 (NOK) and an OSCC cell line with SK2 knockdown (HN13-sh-SK2).

Suppressed or increased miRNAs are involved in the progression of multiple cancers, including oral cancer. Moreover, miRNAs can regulate the DNA damage response.^11,23^ Our previous findings reported that increased SK2 can downregulate miR-205, leading to an aggressive profile in oral carcinoma^15^

MiR-205 plays different roles in the cellular context and is being investigated as a cancer biomarker for both diagnostic and prognostic purposes.^24^ In various types of cancer, such as gastric, prostate, and glioblastoma, miR-205 has been discussed as acting as a tumor suppressor.^13,25,26^ A recent study demonstrated that increased miR-205 sensitizes the radiation response in prostate cancer cells.^27^ Furthermore, in breast cancer cells, miR-205 enhances the chemosensitivity to docetaxel.^13^

In a xenograft model, miR-205 was found to enhance the effect of radiation and suppress DNA damage repair.^28^ Our analysis aimed to overexpress miR-205 in both the NOK-SK2 and HN12-SK2 cell line models to determine if this miRNA could sensitize SK2 cells to DNA damage. We demonstrated that miR-205 can sensitize cells with high SK2 levels to DNA damage by reducing the number of EMT markers and miR-205 targets, including ZEB2 and STAT3. It diminishes stem cell properties and clonogenic potential after UV exposure. In renal cancer, ZEB2 has been shown to be a target of miR-205, which is described as a tumor suppressor.^29^ Additionally, in ovarian cancer cells, ZEB2 has been identified as a target of miR-205.^30^

Furthermore, our protein analysis indicates that miR-205 may recover p53 protein levels by regulating MDM2 expression, which controls p53 stability through ubiquitin-mediated degradation. It has been shown that MDM2 is a target of miR-205.^20^ Surprisingly, HN12 cells, which have undetectable p53 protein, exhibited an increase in p53 protein levels after UV exposure in miR-205-overexpressing cells. Our data suggest a mechanism by which miR-205 influences p53 function. It could be explored as a new strategy to overcome resistance caused by the overexpression or amplification of MDM2.

We also evaluated the miR-205 response in spheres and colonies after cisplatin treatment. Cisplatin is one of the most widely used chemotherapeutic agents in HNSCC, known for causing DNA damage.^31^ Interestingly, similar UV exposure results were observed after cisplatin treatment, showing that miR-205 can sensitize SK2 resistance to DNA damage in both UV exposure and cisplatin treatment.

In conclusion, our study reveals, for the first time, a mechanistic action of SK2 and miR-205 in regulating DNA damage in HNSCC. Perhaps combining miR-205 with DNA damage agents may be a novel strategy for treating HNSCC (Figure 5).

**Figure 5:**
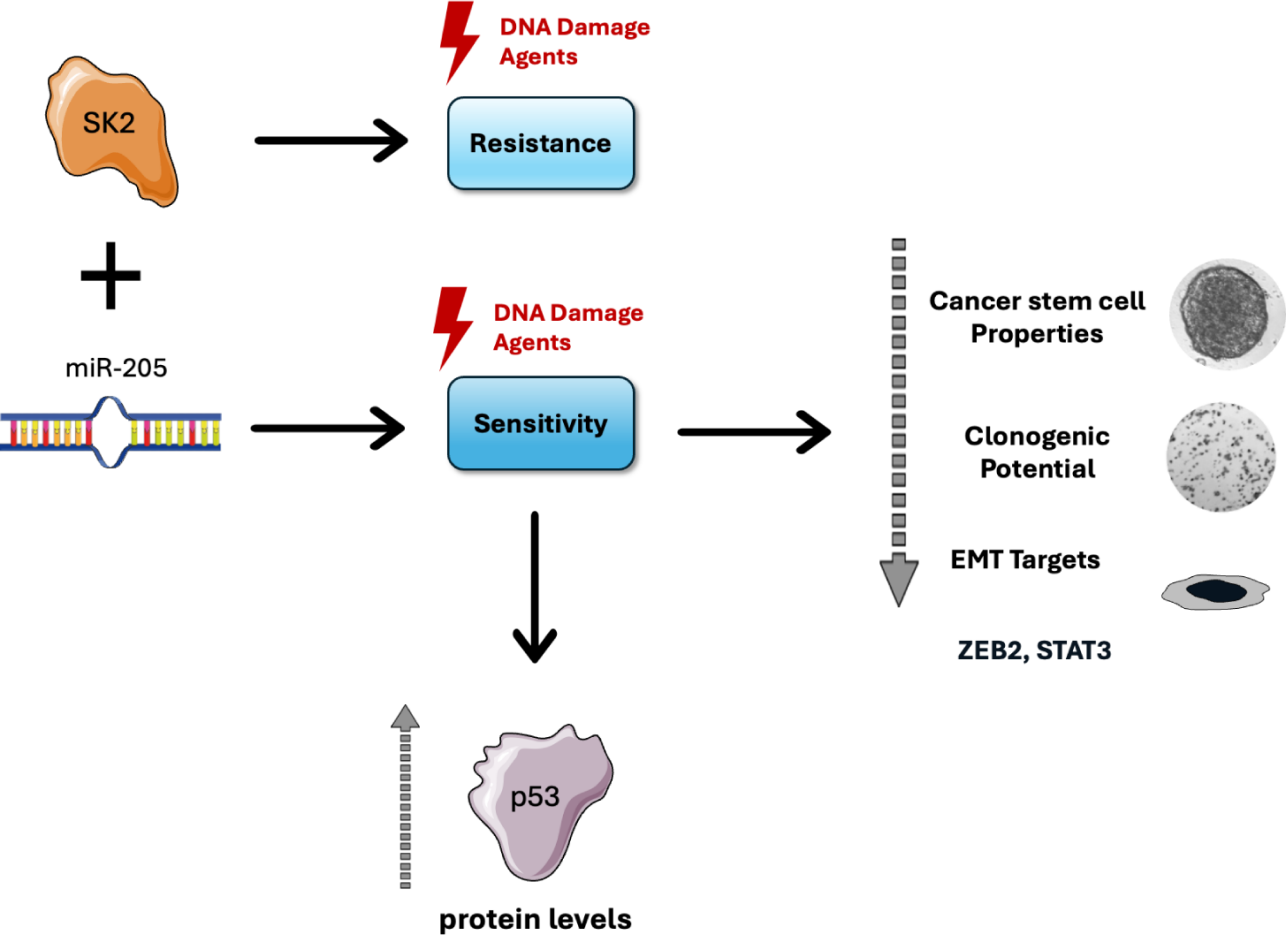
Schematic representation showing the role of miR-205 and the SK2 axis in resistance to genotoxic agents. This schematic illustrates the role of the miR-205/SK2 axis in modulating resistance to DNA damage in oral cancer. SK2 promotes resistance to genotoxic agents and downregulates miR-205. Elevated levels of miR-205, however, inhibit SK2-mediated resistance, reducing the self-renewal capacity of cancer stem cells, clonogenic potential, and epithelial-mesenchymal transition (EMT) targets, and increasing p53 levels, ultimately sensitizing cells to UV and cisplatin. Adapted from Servier Medical Art, licensed under CC BY 3.0 (https://smart.servier.com).

## Methods

### Cell Lines and Cell Culture

The human oral squamous cell carcinoma (HN12 and HN13)^32^ and nontumor human oral keratinocyte cell lines (NOK-Ø)^33^ were previously modified to overexpress (NOK-SK2 and HN12-SK2) or reduce the levels of SK2 (HN13-sh-SK2) using a lentivirus vector or an empty vector (control), as described by refs. 17,18,32,33 . *SK2* expression was measured by qRT-PCR using TaqMan probes: *SK2* - Hs01016543 and the endogenous control *ACTB -* Hs9999999903. The 2^–ΔΔCT^ method was used to calculate the relative expression.^34^ The LMSCC03 primary cell line was established in-house and originated from a tongue squamous cell carcinoma of an 85-year-old female (Human Research Ethics Committee of the School of Pharmaceutical Sciences of Ribeirão Preto-USP, CAAE: 90532418.6.0000.5403). All cells were cultured in DMEM (Sigma-Aldrich, USA), supplemented with 10% (v/v) fetal bovine serum (FBS) (Gibco, Life Technologies, USA), 100 μg/mL penicillin, and 100 μg/mL streptomycin (Sigma-Aldrich, USA), and incubated at 37 °C in a humidified atmosphere of 5% CO_2_.

### miRNA Transfection

To overexpress miR-205, the cells NOK-SK2, HN12-SK2, and LMSCC03 were transfected with pcDNA 3.1/His (Invitrogen) (empty vector) and pcDNA3.2/V5-has-miR-205 plasmid (Addgene #26312). The cells were transfected using PolyJet DNA In Vitro Transfection Reagent (SignaGen Laboratories) and selected with G418 (Sigma-Aldrich no. G818) for 12 days. The relative levels of miR-205 were measured by qRT-PCR using the TaqMan assay: miR-205 (#00509) and RNU6B (#001093) as the internal control. The relative expression was calculated using the 2^–ΔΔCT^ method.^34^

### UV Exposure and Cisplatin Treatment

The SK2-knockdown and overexpressing cell lines, as well as those transfected with mimic miR-205, were exposed to ultraviolet (UV) light in a biosafety cabinet (ESCO Class II BSC) for 30 s, followed by incubation at 37 °C and 5% CO_2_. After 24 h, the cells were plated for the assays. Cisplatin (Sigma-Aldrich) was solubilized in DMSO (Sigma-Aldrich, USA), and 5, 10, and 20 μM of cisplatin or vehicle control (DMSO) was added to the cells for the indicated time.

### Orosphere Formation Assay

For the orosphere formation assay, 2 × 10^3^ and 5 × 10^3^ cells (NOK-SK2 and HN12-SK2, respectively), were seeded in a 96-well plate precoated with poly-2-hydroxyethyl methacrylate (Poly-HEMA, Sigma cat# P3932) or grown as single spheres in ultralow attachment 96-well round-bottom plates (Corning, New York, USA) and maintained in an incubator at 37 °C containing 5% CO_2_ for 5 days. The cells were treated with cisplatin or the vehicle control (DMSO) and exposed to UV to determine chemoresistance. Orospheres with diameters greater than 50 μm were counted or captured using an inverted light microscope (Zeiss Axiovert 40) and then seeded into standard culture medium to attach in 24-well plates (Corning, New York, USA) for the observation of adhesion plasticity. Adherent spheres were fixed in methanol for 20 min and stained with crystal violet for 30 min. Images were acquired using the ChemiDoc MP imaging system (Bio-Rad), and the number of adherent spheres and surface areas were determined using ImageJ.^35^

### Clonogenic Assay

A thousand cells exposed to UV or cisplatin were seeded in 12-well plates (Corning) and maintained at 37 °C for 10 days. Colonies were fixed in methanol for 20 min and stained with crystal violet for 30 min. Images were acquired using the ChemiDoc MP imaging system (Bio-Rad), and the number of colonies (>50 cells) was obtained through ImageJ [15].

### Cell Viability

After cells were seeded in 96-well microplates, they were treated with 5, 10, and 20 μM of cisplatin or vehicle control (DMSO) for 48 h. Cell viability was measured using a resazurin assay, which provided a fluorescence readout on a Biotek Synergy HTX microplate reader (BioSPX, Drogenbos, Belgium).

### Protein Extraction and Western Blotting

Cell lysates were prepared using the CelLytic MT buffer combined with a protease and phosphatase inhibitor cocktail (ThermoFisher Scientific). Protein samples were separated by SDS-PAGE and soaked in a transfer buffer, followed by semidry transfer to PVDF membranes. Membranes were blocked with 5% nonfat dry milk (BioRad #1706404), incubated with primary antibodies against STAT3 (Cell Signaling #9139, 1:1000), phospho STAT3 (Cell Signaling #9145, 1:2000), NM23-H1 (Santa Cruz #sc-343, 1:1000), ZEB2 (Aviva #ARP39141_P050, 1:1000), p53Ac (Cell Signaling #2525, 1:1000), p53 (Cell Signaling #2527, 1:1000), SNAIL (Cell Signaling #3895, 1:1000), Calnexin (Cell Signaling #2433, 1:1000), and GAPDH (Cell Signaling #2118, 1:1000), overnight at 4 °C. Membranes were then incubated with secondary antibodies at room temperature. The immunocomplexes were visualized using a SuperSignal West Dura Extended Duration Substrate (ThermoFisher Scientific), and the chemiluminescence was detected with the ChemiDoc MP imaging system (Bio-Rad).

### Statistical Analysis

Data analysis was performed using GraphPad Prism v9 software (USA). A *t*-test was conducted to compare two groups, and to examine significant differences among multiple groups, a one-way analysis of variance test was performed, followed by Tukey’s multiple comparison test. All experiments were repeated three times independently, and the results are expressed as the means ± standard deviation (SD). *p* < 0.05 was considered statistically significant.

## Supplementary Material













## Notes

(PDF)
(PDF)
(PDF)
(PDF)
(PDF)
Figure S1: SK2 gene expression in nontumor human oral keratinocytes and HNSCC cell lines Figure S2: Cell-adhesion spheres assay of NOK-SK2 cells (PDF)
