# Supplementary figures and images for: miR-205 Enhances Sensitivity to Genotoxic Agents in HNSCC Cells and Blocks Sphingosine Kinase 2 Action in Tumorigenicity
Source: ACS Omega. 2025 Dec 11;10(50):61471–9. doi: 10.1021/acsomega.5c06726 (PMC12750202; doi:10.1021/acsomega.5c06726)

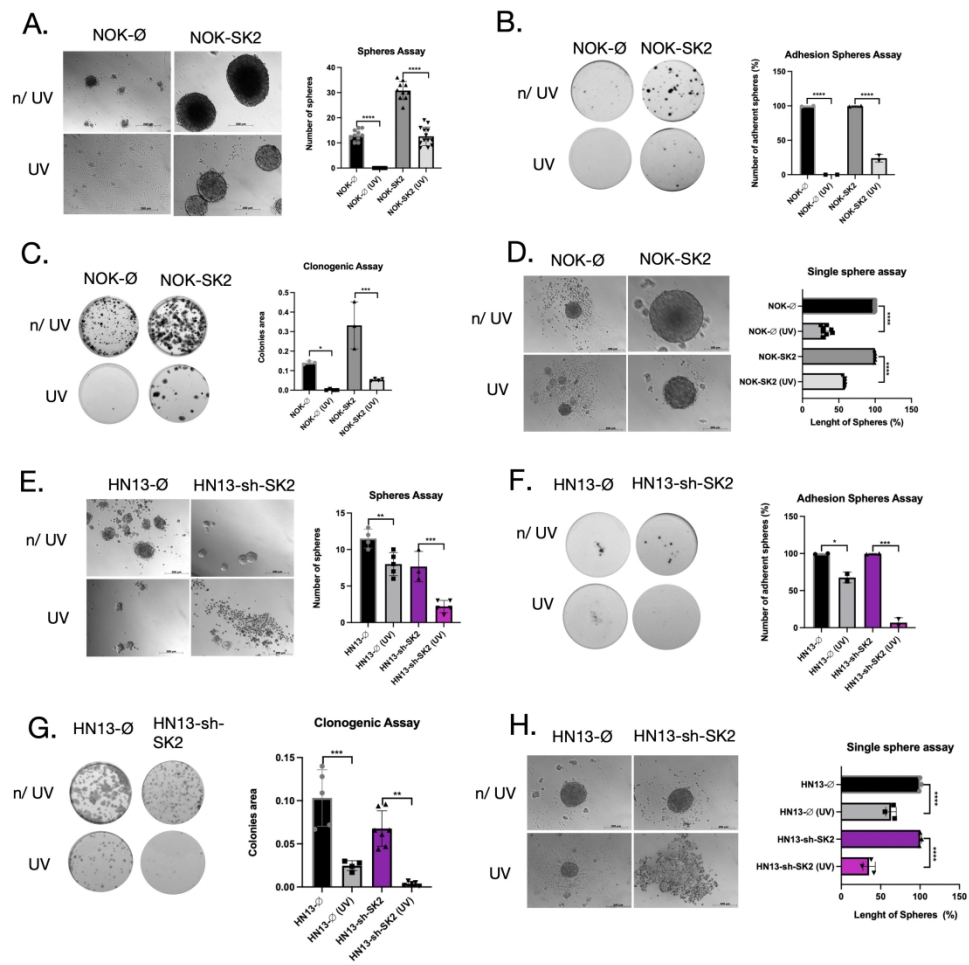

220x216mm (330 x 330 DPI)

Supplement: Supplementary file 1 [file ao5c06726_si_001.pdf]

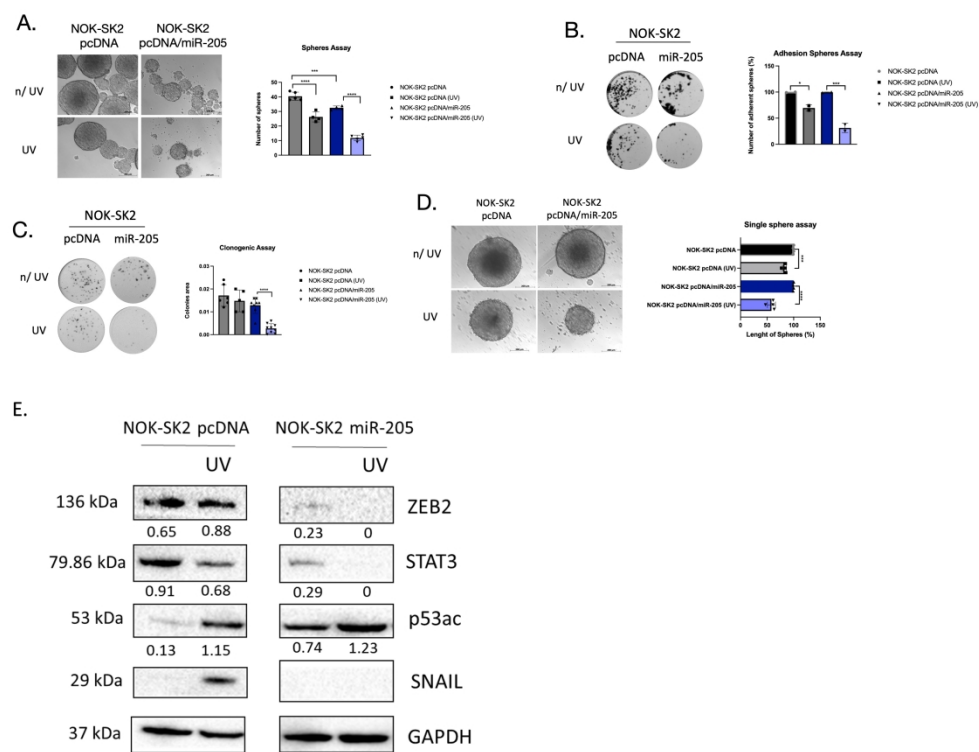

294x223mm (330 x 330 DPI)

Supplement: Supplementary file 2 [file ao5c06726_si_002.pdf]

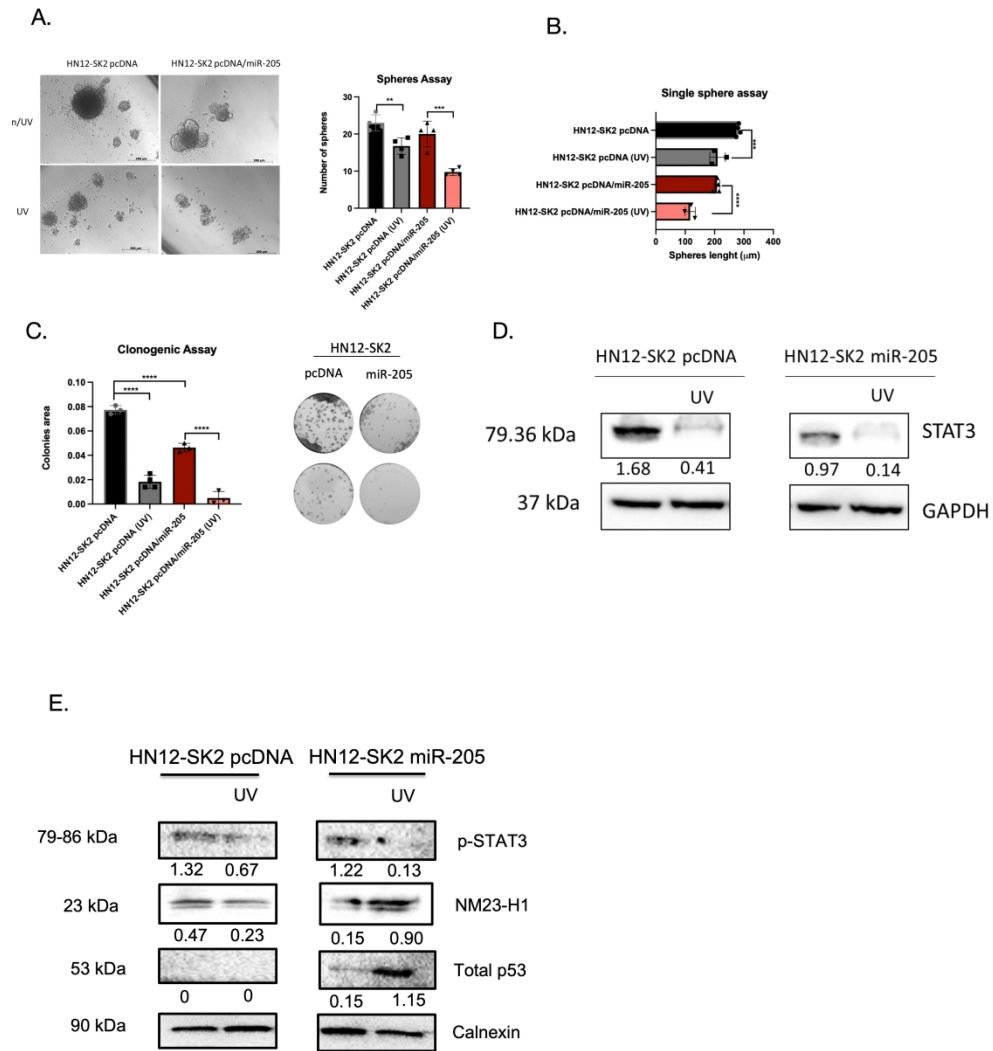

281x297mm (330 x 330 DPI)

Supplement: Supplementary file 3 [file ao5c06726_si_003.pdf]

A.

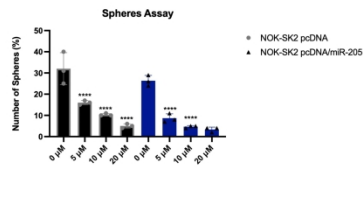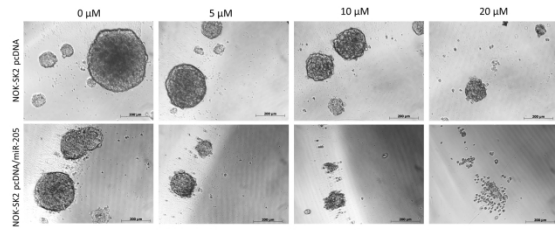

B.

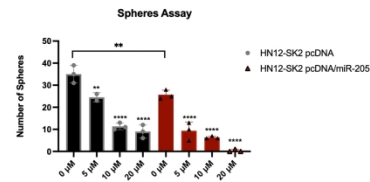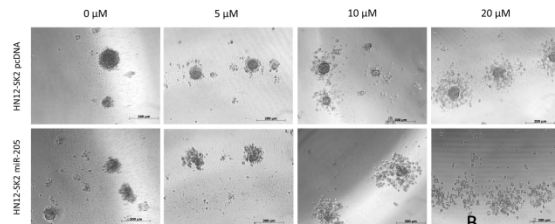

C.

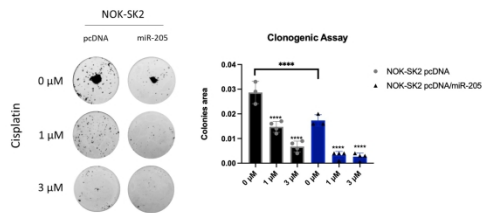

D.

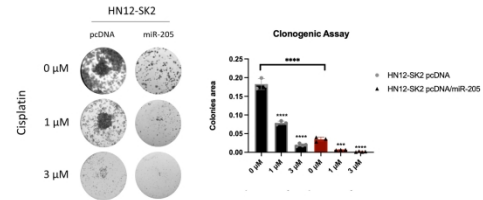

E.

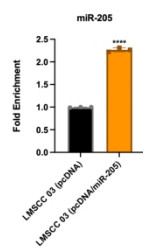

F.

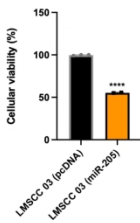

G.

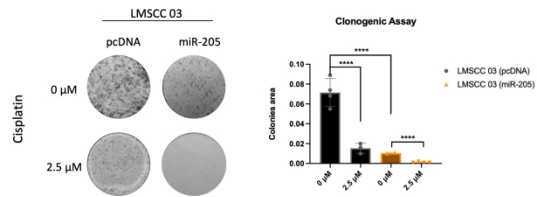

300x335mm (330 x 330 DPI)

Supplement: Supplementary file 4 [file ao5c06726_si_004.pdf]

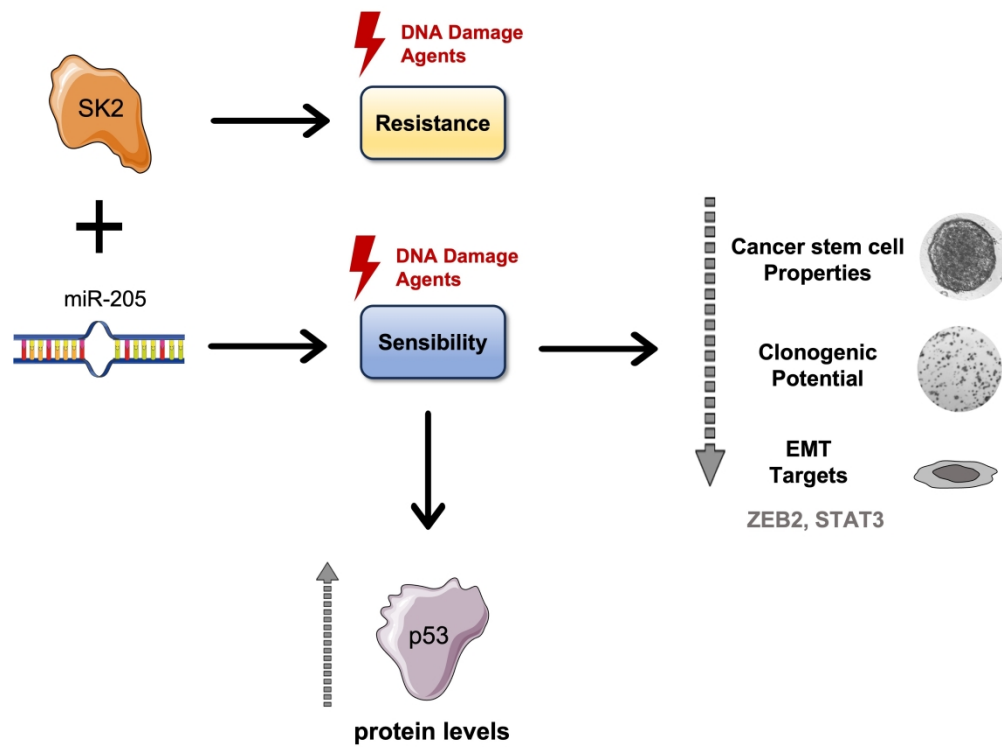

289x219mm (330 x 330 DPI)

Supplement: Supplementary file 5 [file ao5c06726_si_005.pdf]
