# Supplementary material for: miR-205 Enhances Sensitivity to Genotoxic Agents in HNSCC Cells and Blocks Sphingosine Kinase 2 Action in Tumorigenicity
Source: ACS Omega. 2025 Dec 11;10(50):61471–9. doi: 10.1021/acsomega.5c06726 (PMC12750202; doi:10.1021/acsomega.5c06726)

Supporting Information

MiR-205 Enhances Sensitivity to Genotoxic Agents in HNSCC Cells and Blocks Sphingosine Kinase 2 Action in Tumorigenicity

Thaís Moré Milan[1], Gabriel da Silva[1], Graziella Ribeiro de Sousa[1], and Andréia Machado Leopoldino[1]*

[1] Department of Clinical Analyses, Toxicology and Food Sciences, School of Pharmaceutical Sciences of Ribeirão Preto, University of São Paulo, Av. do Café, s/n, Ribeirão Preto, SP, Brazil, 14040-903.

*Corresponding Author:

Andréia M. Leopoldino, Associate Professor

Email: andreiaml@usp.br

Av. do Café, s/n, Bloco A, Sala 994, Ribeirão Preto, SP, Brazil, ZIP Code: 14040-903

Fax: +55 16 3315-4725

Supplementary Figures

Figure S1.
SK2 gene expression in non-tumor human oral keratinocytes and HNSCC cell lines. A–C Cells with SK2 overexpression (NOK-SI and HN12) or knockdown (HN13) were compared to the mock control using qRT-PCR. *P<0.05, **P<0.01.

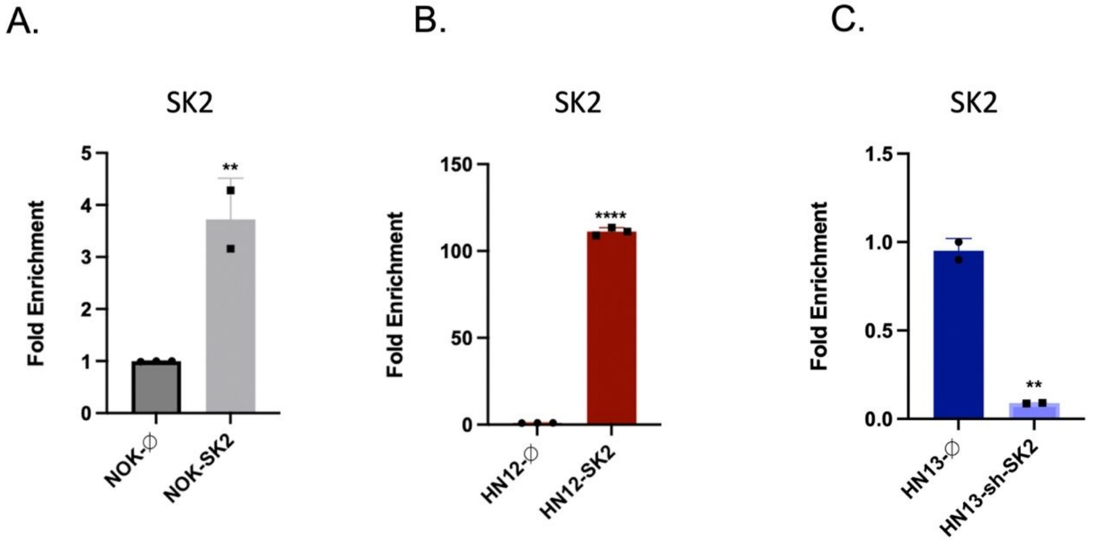

Figure S2.
Cell-adhesion spheres assay of NOK-SK2 cells. A) Adhesion spheres assay on NOK-SK2 cells compared to the mock control. B) Adhesion spheres assay on NOK-SK2 cells with miR-205 overexpression compared to the mock control (pcDNA). *P<0.05.

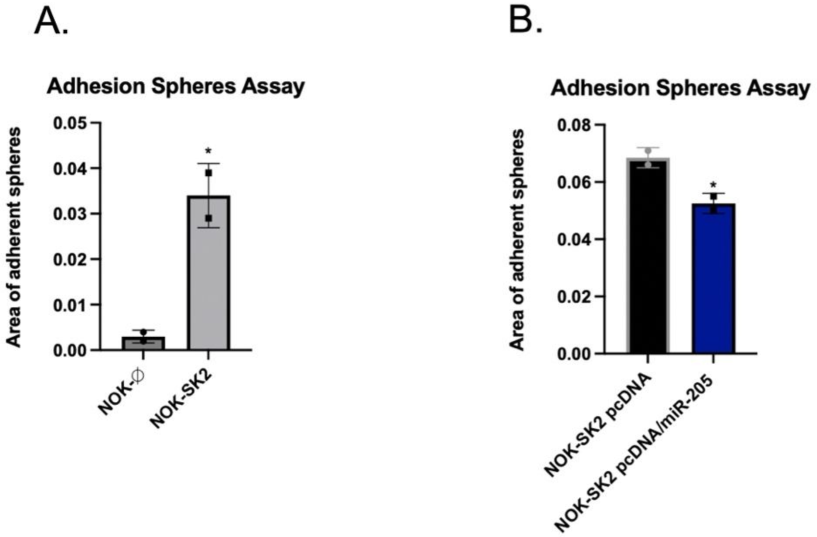

Supplement: Supplementary file 6 [file ao5c06726_si_006.pdf]
